# Repair of anomalous origin of the left coronary artery from the pulmonary artery (ALCAPA) by sinus pouch technique

**DOI:** 10.1186/s13019-022-02100-5

**Published:** 2023-03-04

**Authors:** Masaru Kumae, Mitsuru Aoki, Ikuo Hagino, Hiroshi Koshiyama, Takahiro Ito, Hironobu Nishiori

**Affiliations:** Department of Cardiovascular Surgery, Chiba Children’s Hospital, Chiba, Japan

**Keywords:** Anomalous origin of the left coronary artery from the pulmonary artery, Sinus pouch technique, Infant, Mitral valve regurgitation

## Abstract

A 5-month-old girl, weighing 5.3 kg, diagnosed ALCAPA underwent emergency surgery. The left coronary artery (LCA) originated from the posterior pulmonary artery (PA), and the left main trunk (LMT) was very short (1.5 mm), with moderate level of mitral valve regurgitation (MR). The distance from the origin to the pulmonary valve (Pv) was also short. A free extension conduit was created using adjacent sinus Valsalva flaps and implanted in the ascending aorta to avoid distortion of the coronary artery and the Pv.

ALCAPA causes myocardial ischemia, which leads to impaired cardiac function and MR. Various methods of coronary artery grafting have been reported, and necessity of intervention for MR has been discussed. To the best of our knowledge, there is no report of ALCAPA with coronary artery implantation by applying sinus pouch technique [1], which has been reported in transposition of the great arteries.

## Case report

**Fig. 1:**
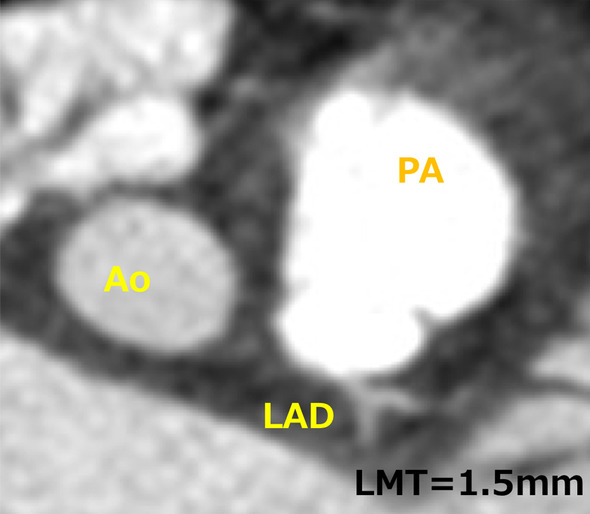
Pre-operative CT

A 5-month-old girl weighing 5.3 kg with no significant medical history had been diagnosed after worsening of heart failure symptoms. Electrocardiogram (ECG) revealed ST elevation at V1-2, negative T at I, II, AVF, V5-6. BNP was 3379 pg/ml. Echocardiography (Echo) showed LCA arising from right posterior sinus of the pulmonary artery (PA) near the valve annulus, left ventricular enlargement (left ventricular end-diastolic volume (LVEDV) was 493% of normal(%N)) [2], decreased cardiac function (left ventricular ejection fraction (LVEF) was 15%), and a moderate level of mitral regurgitation (MR) from the commissures. Computed tomography (CT) demonstrated a short LMT (1.5 mm), and the LCA orifice far from the aorta near the posterior facing commissure (Fig. 1). Operation was scheduled on an emergency basis.

**Fig. 2:**
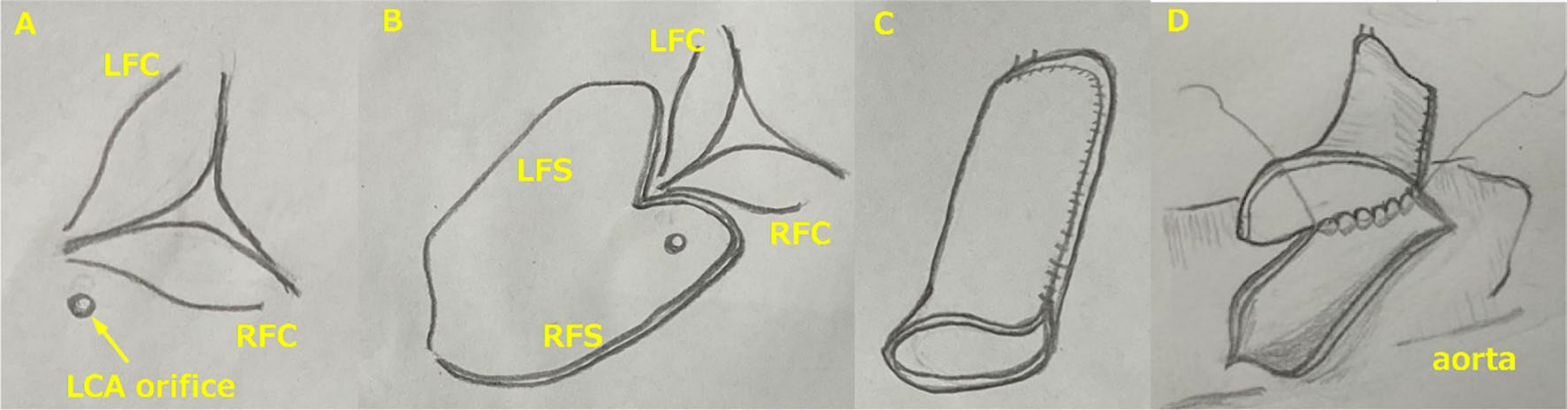
Surgical observations (schema). **A** LFC: left facing cusp, RFC: right facing cusp, LCA: left coronay artery. **B** LFS: left facing sinus, RFS: right facing sinus, LFC: left facing cusp, RFC: right facing cusp. **C**, **D**: see text

The operation was performed by midline sternotomy and under cardiopulmonary bypass with cardioplegia. PA was transected at the bifurcation. The coronary orifice was found in the right facing sinus near the posterior commissure (Fig. 2A). The coronary orifice was detached with a large cuff of two posterior Valsalva sinuses (Fig. 2B). By suturing the right and left parts of the Valsalva sinus flap, the LMT extension was created (Fig. 2C). Anastomosis was made between the end of the Valsalva sinus pouch and an inverted L-shaped incision in the ascending aorta (Fig. 2D). The defect in the posterior sinuses of the PA trunk was supplemented with autologous pericardial patch. The facing commissure of the PA valve was suspended on patch. Kay-Reed annuloplasty was placed at the anterolateral and posteromedial commissures of mitral valve. On postoperative day three, delayed sternal closure was performed. The patient was discharged on aspirin, warfarin, diuretics, enalapril, and carvedilol after 97 days. The LVEDV was 432%N and the LVEF was 23%, and MR was moderate by Echo at the time of discharge.

**Fig. 3:**
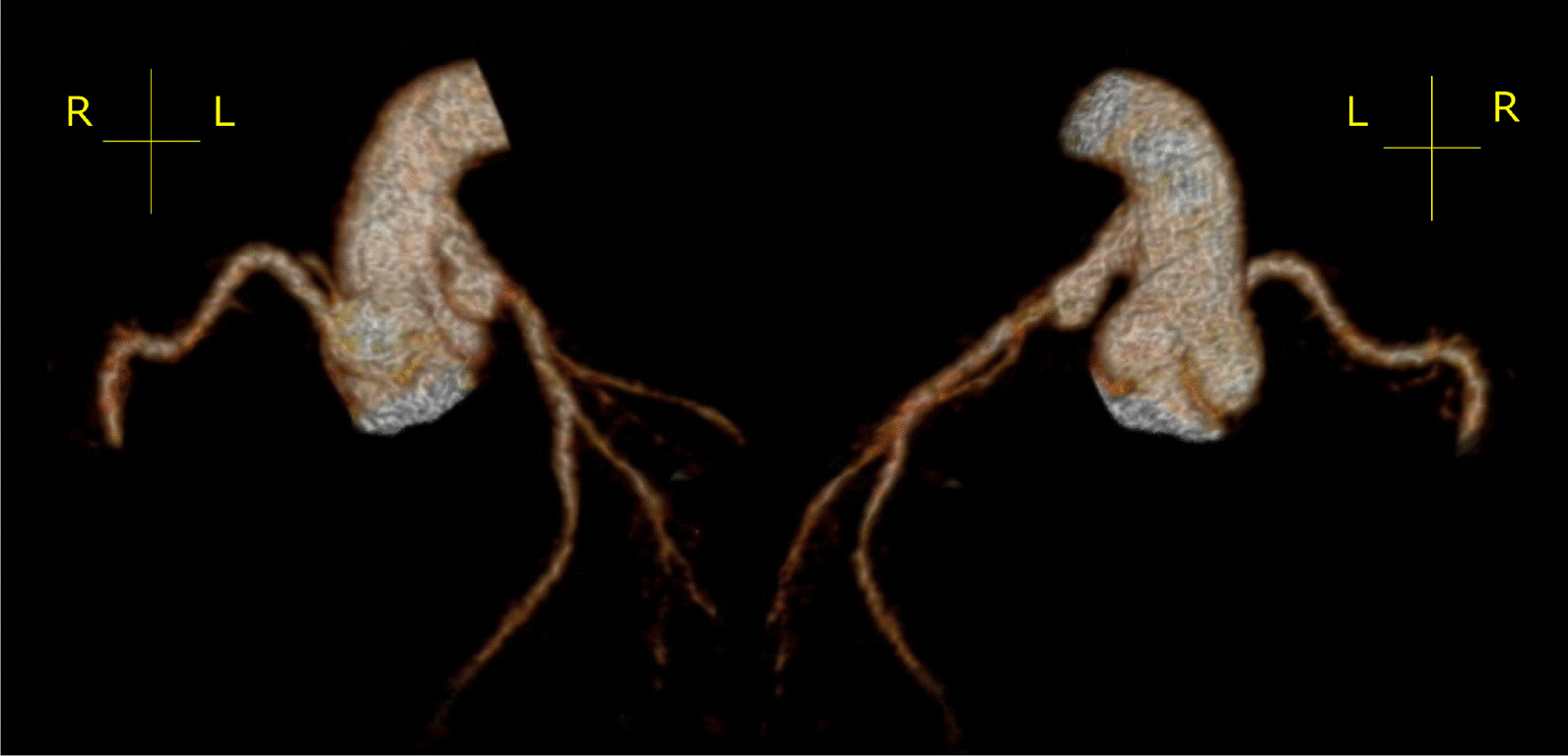
Post-operative CT

Eight months after the surgery, CT indicated a smooth coronary route with no obstructions (Fig. 3). ECG revealed without ST-T changes. BNP was 49 pg/ml. One year after the surgery, Echo showed improvement of left ventricular enlargement (LVEDV was 179%N) and cardiac function (LVEF was 63%). Residual MR was mild-moderate levels from the anterolateral commissure. Catheterization demonstrated a smooth coronary route with no obstructions, LVEDV of 195%N, LVEF of 71%, and Sellers II MR.

Direct coronary implantation to the aorta is the first choice of procedures in ALCAPA, however, several reports of creating a tube extension with a pulmonary artery. Herein successful application of sinus pouch technique [1], reported in transposition of great arteries, in a case of infant-type ALCAPA was described. Various factors such as location of the coronary artery orifice, distance from the aorta, and length of the LMT should be taken in account in choosing a method of coronary artery reconstruction. This technique is advantageous in that it requires less dissection, gives no tension nor torsion to the coronary artery thereby avoiding distortion, and a shorter suture line compared to other conduit methods thereby reducing the risk of bleeding. However, there may be a risk of thrombosis due to a caliber change between the pouch and the LMT, and attention should be paid to anticoagulation and follow-up. The causes of MR have been reported to include papillary muscle dysfunction by ischemia and annular enlargement caused by left ventricular dilation [3]. Rationale of surgical intervention to the MR at the initial operation is disputable. Cons suggested it unnecessary because remodeling was expected to improve regurgitation in the remote phase [4] and pros suggested that addition of relatively simple procedure, such as the Kay-Reed technique helped good control of the regurgitation in the remote phase [^3^]. We believe that active annular reduction facilitates ventricular remodeling in cases of regurgitation level greater than mild.

## Data Availability

The datasets used and/or analysed during the current study are available from the corresponding author on reasonable request.
